# Investigation of antibiotic resistance and biofilm formation ability of *Acinetobacter baumannii* isolated from urinary catheters

**DOI:** 10.12669/pjms.40.11.8754

**Published:** 2024-12

**Authors:** Azka Mumtaz, Unsa Saleem, Muhammad Arif, Nayab Batool

**Affiliations:** 1 Azka Mumtaz, Institute of Microbiology, University of Agriculture, Faisalabad, Pakistan; 2 Unsa Saleem, Institute of Microbiology, University of Agriculture, Faisalabad, Pakistan; 3 Muhammad Arif Livestock and Dairy Development Department, Quetta, Pakistan, Institute of Microbiology, University of Agriculture, Faisalabad, Pakistan; 4 Nayab Batool Center for Advanced Studies, Agriculture & Food Security (CAS AFS), University of Agriculture, Faisalabad, Pakistan, Institute of Microbiology, University of Agriculture, Faisalabad, Pakistan

**Keywords:** *Acinetobacter baumannii*, Multidrug-resistant, Urinary catheters, Biofilm

## Abstract

**Objective:**

Current research aims to monitor the prevalence of *Acinetobacter baumannii* (*A. baumannii)* in healthcare facilities due to the development of resistance to antimicrobials. The study aimed to elucidate the interplay between antibiotic resistance and biofilm formation, two key factors contributing to virulence of bacteria.

**Methods:**

This study was conducted in One Health Laboratory (OHL) at Center for Advanced Studies, Agriculture and Food Security (CAS AFS), and institute of Microbiology University of Agriculture, Faisalabad within six months (Feb-Aug 2023). A total of 50 urine catheter samples were obtained from Allied Hospital, Faisalabad. Antibiotic susceptibility testing (meropenem, ampicillin, ceftriaxone, and gentamicin) was conducted to determine the resistance profiles of the isolates in accordance with the Clinical and Laboratory Standards Institute (CLSI) guidelines. Additionally, the biofilm formation ability of the isolates was assessed using crystal violet staining.

**Results:**

Out of 50 samples, 13 were positive and were confirmed as multidrug resistant. The investigation of antibiotic resistance revealed a high prevalence of MDR *A. baumannii* strains from urinary catheters. The rate of infection was observed higher in males (77%), patients among the age group 10-25 and above 46 years (38.46%), and those who have been diagnosed with urinary tract infection (46.13%). The observed rate of biofilm formation was strong (62%) followed by moderate (7%), and weak (31%) in all MDR isolates. Carbapenem-resistant isolates exhibit a strong correlation with biofilm formation.

**Conclusion:**

This study concluded that *A. baumannii* isolated from the patients with urinary tract infections had resistance to routinely used antibiotics. The isolates have shown hemolysis pattern (α & γ) and tendency to make biofilms. Moreover, except for ceftriaxone which showed negative correlations, a positive correlation was observed between biofilm biomass and the resistance profile to the remaining three antibiotics.

## INTRODUCTION

*Acinetobacter baumannii* is an opportunistic gram-negative, non-fermenting aerobic pathogen that has the potential to cause life-threatening infections in severely immunocompromised patients with extended period of hospitalization.^1^ To counteract the effects of antibiotics, *A. baumannii* uses a variety of tactics, such as β-lactamase synthesis, aminoglycoside metabolic enzymes, target site modification, and efflux pumps.^2^ This pathogen developed resistance to commercialized antibiotics as multi, extensive & and pan-drugs which is the main reason for nosocomial infections with high mortality and morbidity.^3^ Numerous publications have described outbreaks of *A. baumannii* as a nosocomial pathogen that caused pneumonia, and UTI (urinary tract infection).^4^ The World Health Organization (WHO) designated it as a critical microorganism for which novel treatments are urgently needed.^5^

Initially, the most effective drug against *A. baumannii* was carbapenem but now according to the annual surveillance report of antimicrobial resistance by WHO *A. baumannii* attained resistance against it.^6^ Other drugs are also used to treat bacterial infection including meropenem and imipenem colistin, tigecycline, and sub-lactam but it still requires advancement in infection treatment based on novel drug targets.^7^ Moreover, different resistance mechanisms are detected in its clinical isolates describing the different virulence factors including motility of surface, blood agar hemolysis, and biofilm formation. The major reason for *A. baumannii* causing UTI is the biofilm ability in transurethral catheterization.^8^ The fact that *A. baumannii* can form biofilms highlights the significance of infection prevention and control methods in hospital settings.^9^

Understanding antibiotic resistance patterns will facilitate the selection of appropriate treatment regimens, allowing clinicians to optimize therapeutic strategies and improve treatment outcomes. Additionally, assessing the biofilm formation ability of *A. baumannii* strains will aid in the development of targeted preventive measures and infection control strategies, including catheter design modifications and improved catheter maintenance protocols.

## METHODS

This hospital-based cross-sectional study was conducted on 50 catheter indwelled adult patients in Allied Hospital, Faisalabad. The duration of the study was six months (Feb-Aug 2023). Under aseptic conditions, approximately 2-3 ml of urine was taken from an indwelling catheter. The urine specimens were brought to the laboratory for culture and antimicrobial susceptibility testing.

### Ethical Approval:

This study was performed under ethical consideration and approved by Directorate of Graduate Studies (No.DGS/25109-12, Dated: 14-07-2023). Patients included in the study were of different age groups (≥18-84 years) and had at least one urinary catheter in place for more than 48 hours after being admitted to the hospital or having catheter-associated infection symptoms while patients with urinary catheters in place less than 48 hours were excluded from the study.

### Sample processing:

All samples were stored at 4°C, and bacteria were grown overnight at 37°C on MacConkey agar for 18-24 hours aerobically. Isolated colonies were streaked on blood agar plates overnight. Bacterial isolates were identified down to the species level by using standard biochemical tests.^10^

### Antibiotic susceptibility test: 

The disk diffusion method was used to confirm the presence of MDR *A. baumannii* by testing its susceptibility to different antibiotics [meropenem (10μg), ampicillin (30μg), ceftriaxone (30μg), and gentamicin (10μg)] (Oxoid™). The interpretation of the disk diffusion method is according to CLSI guidelines.^11^

### Biofilm assay:

To determine the biofilm formation ability of isolates, the crystal violet staining method was used (3n). Briefly, aliquots of bacterial culture measuring 100 μl with an OD_600_ value of 0.1 were placed in 96 well microtitration plate. Following 37°C incubation for 48 hours, plates were washed with phosphate buffer saline and stained with 0.02% of crystal violet for 10 minutes at room temperature and washed twice to remove extra dye. After drying the plates overnight, the crystal violet was dissolved by adding 200 μl of ethanol: acetone (1:5) for 10 minutes. Finally, the mixture was transferred to a fresh plate and reading was taken at OD_580_ using UV-visible spectrophotometer.^12^

### Statistical analysis:

A test of significance in the distribution profile of positive bacterial samples with respect to age and gender was carried out using the chi-squared test. The relationship between biofilm formation and antibiotic susceptibility was analyzed by the Wilcoxon rank-sum test. All analyses were carried out with one-way ANOVA. Categorical variables between more than two groups were tested, and *p* values of ≤ 0.05 indicated statistically significant.^13^

## RESULTS

Colonies (yellowish & pinkish) and (greyish & whitish) were seen after streaking of A. baumannii following overnight growth on MacConkey agar and sheep blood agar at 37°C. Gram-negative rods were observed under a compound microscope (100X). Further confirmation was done by biochemical test. Isolates were found to be negative for indole and Voges- Prausker test, and positive results were obtained for methyl red, Simmon citrate utilization, catalase and hemolysis test (α, γ hemolysis).

Out of 50 samples, 13 (26%) samples showed positive results for *A. baumannii*. According to statistical analysis, rate of infection was higher in males (77%) as compared to females (23.07%) which was considered significant (*p*<0.05). The rate of infection among the age groups 10-25 years and above 45 years was observed higher (38.46%) as compared to the patients among the age group 25-45 years which was 23.07%. Patients who have urinary tract infections were more prone to infection with A. baumannii as the rate of occurrence was observed higher (46.13%) followed by the surgical (23.07%), ICU (15.40%) and kidney ward (15.40%) (Table-I).

**Table-I:** Distribution profile of positive bacterial isolates.

Demographic and clinical categories	M.D.R A. baumannii (N=50 samples)		p value
	*+ve isolates n=13 (26%)*	*-ve isolates n=37 (74%)*	
***Gender***			0.0365*
*Male*	10 (77)	16 (43.24)	
*Female*	3 (23.07)	21 (56)	
***Age group***			0.4321, NS
(10-25) years	5 (38.46)	11 (30)	
(25-45) years	3 (23.07)	16 (43.24)	
Above 46 years	5 (38.46)	10 (27)	
***Wards***			0.8840, NS
Surgical ward	3 (23.07)	5 (13.51)	
I.C.U	2 (15.40)	12 (27.10)	
Kidney ward	2 (15.40)	8 (16.12)	
Urology ward	6 (46.13)	22 (43.27)	

**Fig.1:**
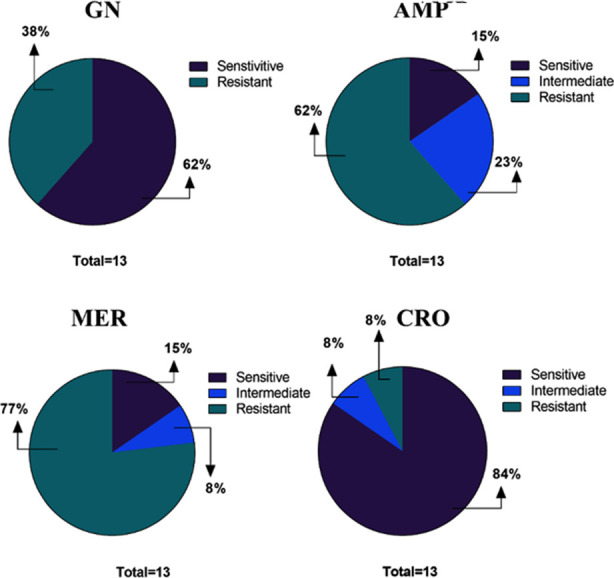
Among the 13 test isolates, resistance to meropenem (77%) was the most common, followed by resistance to ampicillin (62%), gentamycin (38%), and ceftriaxone (8%).

Out of 13 positive isolates 10 were found to be meropenem resistant, two showed sensitivity and one was intermediate resistant. For ceftriaxone, there were 11 sensitive, one resistant, and one intermediately resistant isolate. Gentamycin sensitivity was found in eight isolates, while resistance was found in five isolates. For ampicillin, eight isolates were resistant, one isolate was intermediate resistant, and two isolates showed sensitivity (Fig.1).

All 13 positive samples showed an OD value of greater than 0. The strong biofilm formation measured was 3.85 while the weak biofilm observed was 0.43.

To determine whether biofilm formation is correlated with any antibiotic resistance, biofilm formers with different resistance profiles for the four antibiotics were compared (Table-II).

**Table-II:** Interaction between degree of biofilm formation and antibiotics.

Sr. No	Positive Isolates	Antibiotics				Biofilm

		Ampicillin	Gentamycin	Meropenem	Ceftriaxone	
1	AB5	R	S	S	S	Weak
2	AB6	R	S	R	S	Weak
3	AB7	R	R	R	R	Strong
4	AB9	S	S	R	S	Moderate
5	AB15	R	R	R	S	Strong
6	AB24	R	R	R	S	Strong
7	AB25	R	S	R	R	Strong
8	AB29	R	S	R	S	Weak
9	AB31	R	R	R	S	Strong
10	AB37	S	R	R	S	Strong
11	AB40	R	S	S	S	Weak
12	AB43	R	S	R	S	Strong
13	AB47	R	S	R	S	Strong

**Fig.2:**
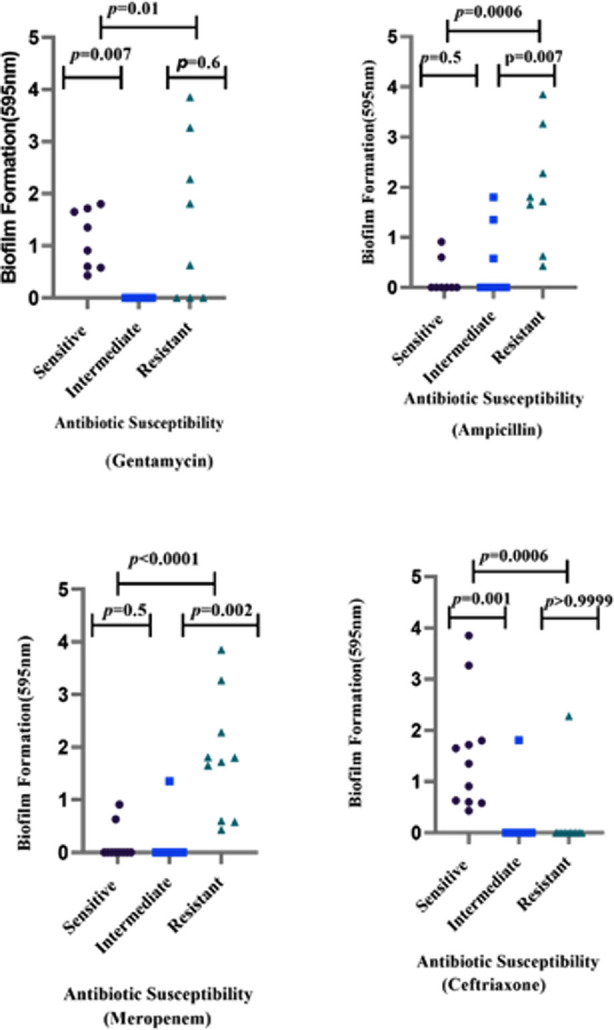
Except for ceftriaxone which showed negative correlations, a positive correlation was observed between biofilm biomass and the resistance profile to the remaining three antibiotics.

According to results, the resistant isolates are prone to form stronger biofilm as compared to susceptible ones. The resistant isolates tend to form stronger biofilm as compared to the susceptible ones. However, in this study biofilm formation ability was observed in ceftriaxone-susceptible isolates (Fig.2).

## DISCUSSION

This study aimed to investigate the antibiotic resistance patterns and biofilm formation ability of *A. baumannii* strains isolated from urinary catheters. The results revealed an average prevalence of *A. baumannii* among the urinary catheter samples, with 13 isolates identified. According to the study, the Urology ward had the highest prevalence of *A. baumannii* at 46.13% isolated from urinary catheters. This is different from a previous study by Shanthi and Sekar, which found isolates from the urinary tract in the ICU ward to be at 25.5%. The prevalence of infections was observed in the surgical ward (23.07%), followed by ICUs (15.40 %) and kidney ward (15.40%).^14^ The rate of infection has been increasing for the past few years.

In the present study, *the rate of A. baumannii* infection was found more prevalent in males (77%) as compared to females (23.07%). There is a similar study which shows a high prevalent rate in males (62%) than in females (38%).^15^ The reason for the high prevalence in males could be smoking, diabetes, alcoholism etc., and in females, the presence of estrogen minimizes the infection.

The occurrence rate was found to be highest (38.46%) among (0-35 years) and above 46 years. These findings are similar to the study conducted in Lahore^16^ which showed the highest (65%) occurrence rate in patients who are less than or upto 20 years. The distribution pattern demonstrates that the age group (1-29 years) and (40-60 years) is more prone to *A. baumannii* infections. This is due to the reason that at younger or older stage there is poor immunity and aging which makes patients more sensitive to *A. baumannii* infections. Based on their antibiotic resistance profile, the prevalence of MDR *A. baumannii* was determined. The study found that the *A. baumannii* isolates (76.9%) were identified as MDR, which means they were resistant to two or more classes of drugs. In our study, the observed meropenem-resistant prevalence rate (77%) is almost equal to the prevalence rate (76.9%).^17^ Carbapenem-resistant Acinetobacter (CRAB) is a critical threat, as recognized by both the Center for Disease Control (CDC) and the World Health Organization (WHO). The continuous prevalence rate is due to unhygienic conditions, healthcare high-cost and prolonged stay in hospitals. However, the *A. baumannii* isolates showed resistance to gentamycin (38%), ampicillin (62%) and sensitive to ceftriaxone (84%). Most isolates exhibited a multidrug-resistant phenotype, making them challenging to treat.

Furthermore, biofilm formation ability was assessed among *A. baumannii* isolates, as biofilms contribute to increased antibiotic resistance and virulence. Among the 13 isolates, the observed rate was stronger (62%) followed by moderate (7%) and weak (31%) biofilm formation which was higher than Cheng-Hong Yang’s findings which showed non-biofilm producers (6.4%), weak biofilm formers (15.6%), moderate biofilm formers (32.4%), and strong biofilm formers (45.4%).^13^ In addition, our study found that the strong biofilm producers tended to be resistant to numerous antibiotics, including gentamycin, ampicillin, and more prominently to meropenem. The results were in accordance with the finding published that MDR bacteria have high tendency to make biofilms.^13^ The investigation of antibiotic resistance revealed a high prevalence of MDR *A. baumannii* strains need multifaceted approach to combat antibiotic resistance and disrupt biofilm formation.

### Limitations:

This study was limited to a single area with limited sample size. Moreover, to identify new treatment for *A. baumanni*i future research should explore a wider range of antibiotics and virulence on genomic level.

## CONCLUSION

This study concluded that *A. baumannii* isolated from the patients with urinary tract infections had resistance to routinely used antibiotics (out of 13 positive isolates 10, 1, 5, and 8 isolates were resistant to meropenem, ceftriaxone, gentamycin and ampicillin respectively). The isolates have shown hemolysis pattern (α & γ) and tendency to make biofilms. Moreover, except for ceftriaxone which showed negative correlations, a positive correlation was observed between biofilm biomass and the resistance profile to the remaining three antibiotics.

### Recommendation:

Current study highlights the urgent need for continued surveillance and monitoring of antibiotic resistance and biofilm formation in *A. baumannii* isolates from urinary catheters in the hospital settings.
